# Age-Related Hearing Impairment (ARHI) Associated with *GJB2* Single Mutation IVS1+1G>A in the Yakut Population Isolate in Eastern Siberia

**DOI:** 10.1371/journal.pone.0100848

**Published:** 2014-06-24

**Authors:** Nikolay A. Barashkov, Fedor M. Teryutin, Vera G. Pshennikova, Aisen V. Solovyev, Leonid A. Klarov, Natalya A. Solovyeva, Andrei A. Kozhevnikov, Lena M. Vasilyeva, Elvira E. Fedotova, Maria V. Pak, Sargylana N. Lekhanova, Elena V. Zakharova, Kyunney E. Savvinova, Nyurgun N. Gotovtsev, Adyum M. Rafailo, Nikolay V. Luginov, Anatoliy N. Alexeev, Olga L. Posukh, Lilya U. Dzhemileva, Elza K. Khusnutdinova, Sardana A. Fedorova

**Affiliations:** 1 Department of Molecular Genetics, Yakut Scientific Centre of Complex Medical Problems, Siberian Branch of the Russian Academy of Medical Sciences, Yakutsk, Russian Federation; 2 Laboratory of Molecular Biology, Institute of Natural Sciences, M.K. Ammosov North-Eastern Federal University, Yakutsk, Russian Federation; 3 Department of Radiology, Republican Hospital #2– Center of Emergency Medicine, Ministry of Public Health of the Sakha Republic, Yakutsk, Russian Federation; 4 Republican Centre of Professional Pathology, Republican Hospital #2– Center of Emergency Medicine, Ministry of Public Health of the Sakha Republic, Yakutsk, Russian Federation; 5 Audiology-Logopaedic Center, Republican Hospital #1– National Medical Centre, Ministry of Public Health of the Sakha Republic, Yakutsk, Russian Federation; 6 Department of Pediatric, Medical Institute, M.K. Ammosov North-Eastern Federal University, Yakutsk, Russian Federation; 7 Department of Normal and Abnormal Anatomy, Operative Surgery with Topographic Anatomy and Forensic Medicine, Medical Institute, M.K. Ammosov North-Eastern Federal University, Yakutsk, Russian Federation; 8 Institute of Foreign Philology and Regional Studies, M.K. Ammosov North-Eastern Federal University, Yakutsk, Russian Federation; 9 Institute of Natural Sciences, M.K. Ammosov North-Eastern Federal University, Yakutsk, Russian Federation; 10 Institute of Humanitarian Research and Indigenous Peoples of the North, Siberian Branch of the Russian Academy of Sciences, Yakutsk, Russian Federation; 11 Institute of Cytology and Genetics, Siberian Branch of the Russian Academy of Sciences, Novosibirsk, Russian Federation; 12 Novosibirsk State University, Novosibirsk, Russian Federation; 13 Department of Genomics, Institute of Biochemistry and Genetics, Ufa Scientific Centre, Russian Academy of Sciences, Ufa, Russian Federation; 14 Department of Genetics and Fundamental Medicine, Bashkir State University, Ufa, Russian Federation; Instituto de Ciencia de Materiales de Madrid – Instituto de Biomedicina de Valencia, Spain

## Abstract

Age-Related Hearing Impairment (ARHI) is one of the frequent sensory disorders registered in 50% of individuals over 80 years. ARHI is a multifactorial disorder due to environmental and poor-known genetic components. In this study, we present the data on age-related hearing impairment of 48 heterozygous carriers of mutation IVS1+1G>A (*GJB2* gene) and 97 subjects with *GJB2* genotype wt/wt in the Republic of Sakha/Yakutia (Eastern Siberia, Russia). This subarctic territory was found as the region with the most extensive accumulation of mutation IVS1+1G>A in the world as a result of founder effect in the unique Yakut population isolate. The *GJB2* gene resequencing and detailed audiological analysis in the frequency range 0.25, 0.5, 1.0, 2.0, 4.0, 8.0 kHz were performed in all examined subjects that allowed to investigate genotype-phenotype correlations between the presence of single mutation IVS1+1G>A and hearing of subjects from examined groups. We revealed the linear correlation between increase of average hearing thresholds at speech frequencies (PTA_0.5,1.0,2.0,4.0 kHz_) and age of individuals with *GJB2* genotype IVS1+1G>A/wt (*r_s_* = 0.499, *p* = 0.006860 for males and *r_s_* = 0.427, *p* = 0.000277 for females). Moreover, the average hearing thresholds on high frequency (8.0 kHz) in individuals with genotype IVS1+1G>A/wt (both sexes) were significantly worse than in individuals with genotype wt/wt (p<0.05). Age of hearing loss manifestation in individuals with genotype IVS1+1G>A/wt was estimated to be ∼40 years (*r_s_* = 0.504, *p* = 0.003). These findings demonstrate that the single IVS1+1G>A mutation (*GJB2*) is associated with age-related hearing impairment (ARHI) of the IVS1+1G>A carriers in the Yakuts.

## Introduction

Age-related hearing impairment (ARHI), or presbyacusis, is a common sensory disorder characterized by bilateral sensorineural hearing loss more evident at high frequencies [1], [2]. According to some data, hearing acuity decrease occurs in one out of 25 persons at age older than 45 [3], and about 50% of population over 80 years has a significant hearing impairment [4]. ARHI is a multifactorial disease [1], [5], but the age of disease onset has not been determined. Recently, using a genomewide analysis two loci 8q24.13-q24.22 (ARHI 1, OMIM 612448) [1], [6], 3p26.1-p.25.1 (ARHI 2, OMIM 612976) [1], [7], [8] were linked with this disease.

Genes already known to be associated with hearing impairment are not investigated enough for the presence of genetic associations with ARHI. Mutations in *GJB2* gene coding protein connexin 26 (Cx26) in homozygous and compound-heterozygous states are responsible for a significant part of inherited autosomal recessive forms of hearing loss in different populations [9]. However, several studies suggest that even single mutations in *GJB2* gene (in heterozygous state) have influence on hearing function [10]–[16]. These studies of hearing thresholds in carriers of recessive mutations in *GJB2* gene – c.35delG, p.Met34Thr, c.167delT (most frequently registered in Europe), and c.235delC single mutation (the study was carried out in China) – had controversial results [10]–[13], [15], [16].

Splice site mutation IVS1+1G>A in *GJB2* gene is relatively rare, and audiological analysis of hearing threshold in carriers of this single mutation has not been performed previously. High rates of IVS1+1G>A mutation (2–11.7%) were found among some indigenous populations of Eastern Siberia, Russia (Yakuts, Dolgans, Evenks and Evens), with the highest carrier frequency (11.7%) in isolated Yakut population [17]. The genetic data revealed a relatively small size of Yakut ancestor population and the strong bottleneck effect in the Yakut paternal line (80% of Y chromosomes of Yakuts belong to one haplogroup – N1c) [18]–[21]. Extremely high rate of IVS1+1G>A mutation in relatively genetic homogenous Yakut population gives a unique opportunity to investigate hearing status in individuals heterozygous for this *GJB2* gene mutation.

The purpose of this study is to analyze age-related hearing alteration in *GJB2* mutation IVS1+1G>A carriers versus individuals with normal *GJB2* genotype in Yakut population.

## Material and Methods

### Sampling design

**Figure 1:**
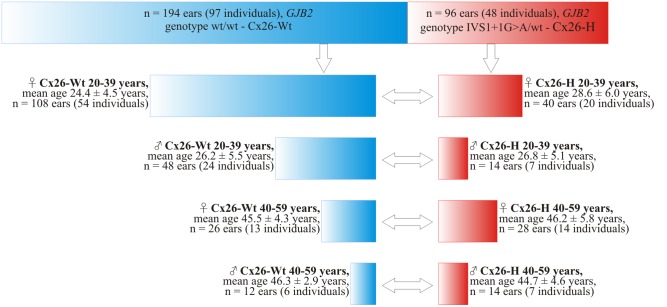
Design of Cx26-H and Cx26-Wt groups. Note: Cx26-Wt (IVS1+1G>A non-carriers) is shown in blue, Cx26-H (IVS1+1G>A carriers) is shown in red; bilateral arrows show the compared subgroups; ♀ – female, ♂ – male.

To form the total sample we invited random volunteers without any complaints on hearing (both in the Yakut Scientific Centre of Complex Medical Problems and during field trips in regions of inhabitance of the Yakutia indigenous population) and informed the potential participants about the aims of upcoming study. We also invited the parents and siblings of deaf children homozygous for the IVS1+1G>A mutation, as they could be the carriers of this mutation. All the volunteers (214 individuals in total) passed otologic-audiological evaluation and genotyping for the *GJB2* gene mutations (spectrum of *GJB2* genotypes is given in Table 1). For the further analysis we selected 145 individuals that matched the following criteria: had *GJB2* genotypes wt/wt or IVS1+1G>A/wt; were older than 20 years old; belonged to Yakut ethnicity (predominantly to the third generation); had no recent history of fever, otitis media, tinnitus; and had no obvious contacts with occupational noise. From those 145 individuals based on genotyping data we formed two groups: Cx26-H – the group included the carriers of single mutation IVS1+1G>A (*GJB2* genotype IVS1+1G>A/wt, 48 individuals or 96 ears); and Cx26-Wt – the group of non-carriers of the IVS1+1G>A mutation (*GJB2* genotype wt/wt, 97 individuals or 194 ears). Subsequently, we stratified these two groups according to age and sex for the purpose of correct comparison (Figure 1).

**Table 1:** Mutation analysis of *GJB2* gene.

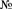	*GJB2* genotype	Relatives of the deaf children with mutations in *GJB2* gene	Random sample from Yakut population	Frequency of *GJB2* genotypes*, %
**1**	**wt/wt**	**4**	**113**	**66.47**
**2**	**IVS1+1G>A/wt**	**35**	**17**	**10.00**
3	IVS1+1G>A/p.Val27Ile	2	2	1.18
4	p.Val27Ile/wt	1	21	12.35
5	p.Val27Ile/p.Val27Ile+p.Thr123Asn	-	1	0.59
6	p.Val27Ile+p.Glu114Gly/wt	-	6	3.53
7	p.Val27Ile+p.Thr123Asn/wt	-	5	2.94
8	p.Met34Thr/wt	-	1	0.59
9	p.Val37Ile/wt	-	3	1.76
10	c.35delG/wt	2	-	-
11	c.360_362delGAG/wt	-	1	0.59
Total number and percentage of *GJB2-*mutations and polymorphisms		40	57	33.53
Total number of individuals		44	170	-
Total number and percentage		214		100

Note: * The frequency of *GJB2* genotypes was calculated in random sample; in bold are *GJB2* genotypes selected for comparative analysis of hearing thresholds.

### Audiological examination

Audiological examination was conducted on all 214 individuals (n = 428 ears). Otologic examination was performed with the «Basic-Diagnostik-Set» (KaWe, Germany), tuning fork standard set. Acoustic impedance was measured with impedance meter (Interacoustics AS, Denmark). Air conduction thresholds were measured on frequencies 0.25, 0.5, 1.0, 2.0, 4.0, 8.0 kHz and bone conduction thresholds – on frequencies 0.25, 0.5, 1.0, 2.0, 4.0 kHz with audiometer «MAICO ST20» (MAICO, Germany) in the same conditions for all participants. The results were assessed separately for each ear on air conduction thresholds as on all measured frequencies, and speech range of frequencies – PTA_0.5,1.0,2.0,4.0 kHz_ separately.

### Mutation analysis of GJB2 gene

A total of 214 genomic DNA samples, which were extracted from leukocytes of peripheral blood, were used for *GJB2* gene mutation analysis. Amplification of the coding (exon 2) and noncoding (exon 1) and flanking intronic regions of *GJB2* gene was conducted with PCR on thermocycler «MJ Mini» (Bio-Rad) using appropriate primers [22]–[25]. The PCR products were subjected to direct sequencing using the same primers on ABI PRISM 3130XL (Applied Biosystems, USA) («Genomics» Core Facility; Institute of Chemical Biology and Fundamental Medicine, Siberian Branch of the Russian Academy of Sciences, Novosibirsk, Russia). The results of mutation analysis of *GJB2* gene are shown in the Table 1.

### Statistical analysis

The results of audiological examination and mutation analysis of *GJB2* gene were summarized in the unified database. Regression and correlation analysis of «age-hearing threshold» association were assessed with Spearman's rank correlation coefficient *r_s_*. Significance of hearing thresholds on measured frequencies was statistically evaluated with the Mann-Whitney U test using software STATISTICA version 8.0. (StatSoft Inc, USA) and Biostatd (McGraw-Hill, Inc.Version 3.03). Differences were statistically significant when *p*<0.05.

### Ethical approval

Written informed consent was obtained from all individuals. This study was approved by the local Committee on Biomedical Ethics of Yakut Scientific Center of Complex Medical Problems, Siberian Branch of the Russian Academy of Medical Sciences, Yakutsk, Russia (Yakutsk, Protocol No 16, April 16, 2009).

## Results

### Correlation analysis of hearing thresholds in PTA_0.5,1.0,2.0,4.0 kHz_ according to age

**Figure 2:**
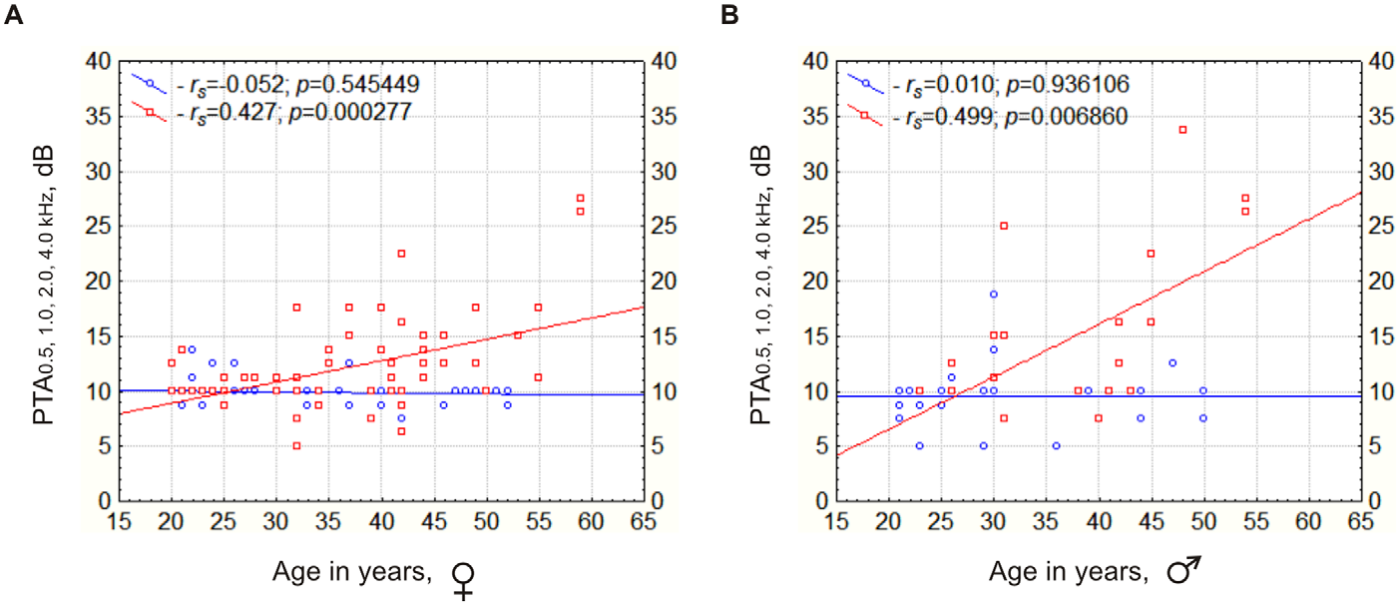
The correlation of increase of hearing thresholds according to age in individuals with genotype IVS1+1G>A/wt. Note: Y-axis – hearing thresholds (dB), X-axis – age in years; *r_s_* – the Spearman's rank correlation coefficient, *p* – statistical significance of Spearman's rank correlation; blue circles – individuals with *GJB2* genotype wt/wt, red squares – individuals heterozygous for mutation (genotype IVS1+1G>A/wt); blue line shows the absence of hearing thresholds correlation with age in individuals with *GJB2* genotype wt/wt, red line – the linear regression of increase in hearing thresholds according to age in individuals with *GJB2* genotype IVS1+1G>A/wt; A – ♀ female, B – ♂ male.

We conducted correlation analysis of «age-hearing thresholds at PTA_0.5,1.0,2.0,4.0 kHz_» in IVS1+1G>A single mutation carriers (group Cx26-H) and non-carriers (group Cx26-Wt), for females and males separately. Statistically significant (*p*<0.05) correlation of increase in hearing threshold according to age was detected in IVS1+1G>A carriers (group Cx26-H: *r_s_* = 0.427, *p* = 0.000277 in females, *r_s_* = 0.499, *p* = 0.006860 in males) and not observed in non-carriers (group Cx26-Wt: *r_s_* = −0.052, *p* = 0.545449 in females, *r_s_* = 0.010, *p* = 0.936106 in males) (Figure 2 A, B).

### The age of onset of hearing impairment in single mutation IVS1+1G>A carriers

To determine onset of presbyacusis manifestation, both studied groups Cx26-H and Cx26-Wt were divided into age cohorts 20–29, 30–39, 40–49, and 50–59 years. Correlation analysis of association «age-hearing threshold in PTA_0.5,1.0,2.0,4.0 kHz_» was performed in these age cohorts (Table 2). Correlation was not observed in individuals of all age cohorts from group Cx26-Wt and in individuals at ages 20–29 and 30–39 years from group Cx26-H. Statistically significant (*p*<0.05) correlation was observed in hearing thresholds of individuals from group Cx26-H at ages 40–49 years (*r_s_* = 0.504, *p* = 0.003), and 50–59 years (*r_s_* = 0.697, *p* = 0.024) (Table 2). Thus, hearing impairment onset was identified as approximately 40 years, and further analysis was conducted in two age subgroups: 20–39 and 40–59 years (for females and males, separately).

**Table 2:** Correlation of increase of hearing thresholds in PTA_0.5,1.0,2.0,4.0 kHz_ according to age in individuals with *GJB2* genotype wt/wt and IVS1+1G>A/wt.

*GJB2* genotype	Parameters	20–29 years	30–39 years	40–49 years	50–59 years
wt/wt	n = ears	128	28	28	10
	*r_s_*	0.036	−0.245	0.159	0.272
	*p*	0.682	0.208	0.417	0.445
IVS1+1G>A/wt	n = ears	28	26	32	10
	*r_s_*	0.228	−0.125	**0.504**	**0.697**
	*p*	0.243	0.542	**0.003**	**0.024**

Note: *r_s_* – the Spearman's rank correlation coefficient; values with statistically significant correlation (p<0.05) of increase of hearing thresholds with age are shown in bold.

### Detailed audiometric analysis of hearing thresholds in IVS1+1G>A carriers and non-carriers

**Figure 3:**
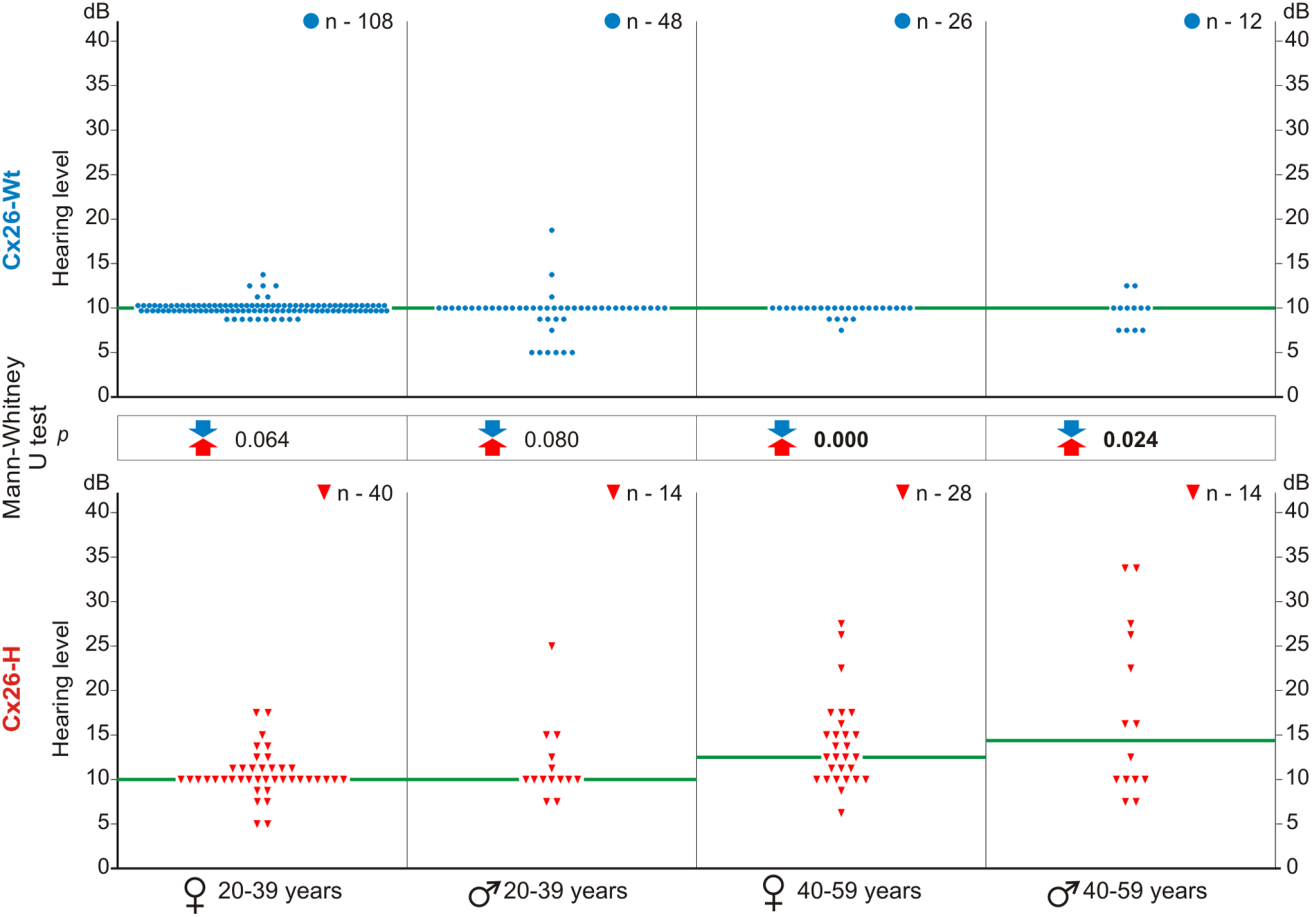
Scatter plots of the hearing thresholds at PTA_0.5, 1.0, 2.0, 4.0 kHz_ of individuals from compared Cx26-Wt and Cx26-H subgroups according to sex and age. Note: Y-axis – hearing level (hearing threshold, dB). X-axis – audiometric parameters of individuals according to sex and age. Blue circle denotes an individual with *GJB2* genotype wt/wt, red delta denotes an individual with *GJB2* genotype IVS1+1G>A/wt; n – number of ears; statistically significant differences (p<0.05) by the Mann-Whitney U test between the compared subgroups are shown in bold; median hearing thresholds are shown by green line. ♀ – female; ♂ – male.

Comparative analysis of hearing thresholds at frequencies of significant speech range (PTA_0.5,1.0,2.0,4.0 kHz_) did not identify differences between groups Cx26-Wt and Cx26-H at age 20–39 years (females *p* = 0.064, males *p* = 0.080). However, statistically significant differences in hearing thresholds were observed at age 40–59 years in both groups Cx26-Wt and Cx26-H (females *p* = 0.000, males *p* = 0.024) (Figure 3).

**Figure 4:**
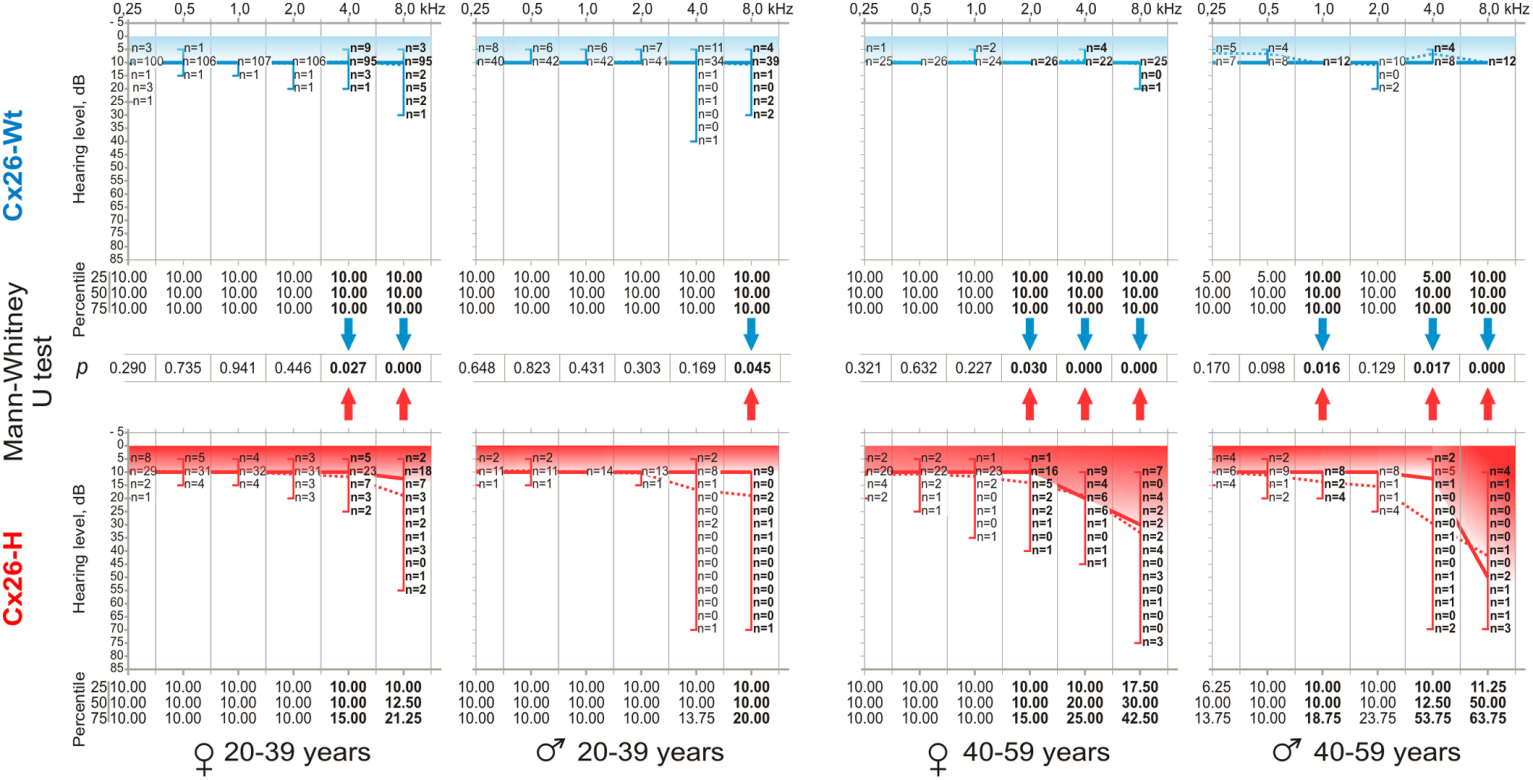
Audiometric parameters at all measured frequencies (0.25, 0.5, 1.0, 2.0, 4.0, 8.0 kHz) of individuals from compared subgroups Cx26-Wt and Cx26-H according to sex and age. Note: Y-axis – hearing thresholds (dB), X-axis – measured frequency (kHz), the audiometric parameters of individuals with *GJB2* genotype wt/wt are shown in blue and of individuals with *GJB2* genotype IVS1+1G>A/wt – in red; n – the number of ears; the frequencies (%) with statistically significant differences (p<0.05) by the Mann-Whitney U test are shown in bold; the arrows show statistically significant differences (p<0.05) by the Mann-Whitney U test; median hearing thresholds are shown by solid line, average hearing thresholds – by dotted line. ♀ – female; ♂ – male.

Following analysis at all measured frequencies (0.25, 0.5, 1.0, 2.0, 4.0, 8.0 kHz) revealed that in group Cx26-Wt averaged audiological profile of females and males aged 20–39 and 40–59 years had a shape of flat curve. Averaged audiological profile of females and males aged 20–39 years in group Cx26-H had a shape of sloping curve, aged 40–59 years – steep-sloping curve in high-frequent range (4.0–8.0 kHz) (Figure 4).

Hearing threshold median at all measured frequencies did not reach 20.0 dB in males and females aged 20–39 years from both groups (Cx26-Wt and Cx26-H) (Figure 4). However, statistically significant (*p*<0.05) differences were observed at frequency 8.0 kHz in both sexes and at frequency 4.0 kHz – in females (Figure 4).

Statistically significant differences (*p*<0.05) in hearing thresholds at frequencies 4.0–8.0 kHz between groups Cx26-Wt and Cx26-H were observed in females and males aged 40–59 years (differences for females were observed starting from frequency 2.0 kHz, for males – from 1.0 kHz). Moreover, median value of hearing threshold was ≥20.0 dB in single mutation IVS1+1G>A carriers at 4.0–8.0 kHz for females, and at 8.0 kHz – for males (Figure 4).

## Discussions

ARHI is multifactorial disorder due to environmental and poor-known genetic components. Studying the contribution of single mutations in *GJB2* gene to ARHI had somewhat controversial results.

Engel-Yeger et al. did not detect difference in mean hearing thresholds between c.35delG mutation – carriers (n = 40 persons) and individuals with normal *GJB2* genotype [11]. However, they found proved differences in otoacoustic emission amplitude between the compared groups [26]. Also, associations between c.35delG heterozygous carrier state and age-related hearing alterations with influence of occupational noise were not shown even on more representative sample (2311 individuals) [13]. It is possible that in these studies the difference in hearing thresholds between carriers and non-carriers was not detected due to the inadequate sample size or due to specific features of the chosen method of analysis.

Moreover, there is a study that detected statistically significant differences in mean hearing thresholds on high frequencies (6.0 and 8.0 kHz) between mutation c.35delG carriers (n = 31) and individuals with normal *GJB2* genotype (n = 29) in Italy, however, this study failed to find a correlation of hearing threshold increase with age [12]. Hearing loss predominantly on high and extra-high frequencies (8.0–16.0 kHz) was found to be significantly more severe in *GJB2* c.35delG heterozygous females in comparison with controls [15]. The recent study of hearing status in carriers of *GJB2* single mutations, c.35delG and p.Met34Thr (c.101T>C), conducted in a UK population from 1991 to 2011 with three-step audiological test of children aged 3, 7 and 11 years (9202 children) showed that hearing thresholds on extra-high frequency (16.0 kHz) in c.35delG and p.Met34Thr (c.101T>C) carriers was significantly worse than in children without *GJB2* gene mutation and tend to increase with age [14].

A recently published study provides the data comparable to our results. The study carried out in China (2011) showed that in c.235delC heterozygous carriers hearing for high-frequency (4.0 and 8.0 kHz) increases at the age of 30–59 years and hearing thresholds in the intermediate frequencies may deteriorate over the age of 40 [16].

Our study provides the first evidence that in a relatively isolated Yakut population the average hearing thresholds at the speech frequencies (PTA_0.5,1.0,2.0,4.0 kHz_) linearly increase (p<0.05) with age in the heterozygous carriers of single site splicing mutation IVS1+1G>A (*GJB2* gene) of both sexes. This correlation is more expressed in males, which probably associated with physiological features and environmental factors. Moreover, audiometric analysis showed that hearing thresholds at high frequency (8.0 kHz) in heterozygous for IVS1+1G>A females and males aged 20–39 years were definitely worse (*p*<0.05) than in non-carriers. We also found that hearing on lower frequencies (1.0–2.0 kHz) was affected in both heterozygous for IVS1+1G>A females and males aged 40–59 years (Figure 4). At the same time, these parameters in non-carriers do not cause concerns about hearing impairment regardless of age and sex. We determined onset of hearing worsening in individuals with genotype IVS1+1G>A/wt as ∼40 years (*r_s_* = 0.504, *p* = 0.003). Thus, we identified that hearing acuity of single mutation IVS1+1G>A carriers on particular frequencies was decreased compared to non-carriers, regardless of sex and age. Splice site mutation IVS1+1G>A, probably leads to a deficiency of normal Cx26 molecules that form intercellular gap junction channels, and this results in impaired recycling of potassium ions in the cochlea with a local organ of Corti intoxication [27]. Thus, carrying the single mutation IVS1+1G>A may cause hearing worsening with ageing.

## Conclusion

Despite the controversial results of previous audiological analysis of the c.35delG mutation in heterozygous carriers from Caucasian populations [10]–[15], the results of this study demonstrate that the single IVS1+1G>A mutation (*GJB2*) is associated with age-related hearing impairment (ARHI) of the IVS1+1G>A carriers among the Yakuts.
